# Toll-like Receptor 4 Modulation as a Strategy to Treat Sepsis

**DOI:** 10.1155/2010/568396

**Published:** 2010-04-14

**Authors:** X. Wittebole, D. Castanares-Zapatero, P. F. Laterre

**Affiliations:** Critical Care Unit, Acute Medicine Department, St Luc University Hospital, Université Catholique de Louvain, Avenue Hippocrate 10, 1200 Bruxelles, Belgium

## Abstract

Despite a decrease in mortality over the last decade, sepsis remains the tenth leading causes of death in western countries and one of the most common cause of death in intensive care units. The recent discovery of Toll-like receptors and their downstream signalling pathways allowed us to better understand the pathophysiology of sepsis-related disorders. Particular attention has been paid to Toll-like receptor 4, the receptor for Gram-negative bacteria outer membrane lipopolysaccharide or endotoxin. Since most of the clinical trial targeting single inflammatory cytokine in the treatment of sepsis failed, therapeutic targeting of Toll-like receptor 4, because of its central role, looks promising. The purpose of this paper is to focus on the recent data of various drugs targeting TLR4 expression and pathway and their potential role as adjunctive therapy in severe sepsis and septic shock.

## 1. Introduction

Despite a decrease in mortality over the last decade, sepsis remains the tenth leading causes of death in western countries and one of the most common cause of death in intensive care units [1]. Between 1979 and 2000, there was an annualised increase in the incidence of sepsis of 8.7 percent, reaching 240.4 per 100000 people in 2000 [2]. Despite progress in better recognition and improved standard of care, mortality still ranges from 30 to 50% in patients with septic shock [3]. Hence, unmet needs for those patients are still present.

About 12 years ago, the discovery of the Toll-like receptor (TLR) unravelled the missing link between endotoxin recognition by LBP and CD14 and the intracellular signalling pathway, leading to the activation and translocation of NF*κ*B to the nucleus, and the subsequent production of proinflammatory cytokines [4–6]. TLRs were first described in Drosophila melanogaster where it functions as a key receptor for dorsoventral polarity during development and is required for immunity against fungal infections [7]. The toll-signalling pathway was shown to have major similarities with the mammalian IL-1 receptor pathway. To date, 10 TLR or pattern-recognition receptors (PRRs) are identified in human and a series of studies have revealed their respective ligands [8–10]. TLRs recognize essential structures expressed by pathogen (collectively referred to as Pathogen Associated Molecular Patterns or PAMPS) as well as endogenous mediators released during tissue damage, independently of infectious state (these mediators referred to as alarmins or Danger Associated Molecular Patterns or DAMPS). The role of TLR and TLR signalling in the pathogenesis and development of sepsis was recently reviewed [11–13]. 

In order to prevent an overwhelming activation of TLR, and its potential side effects, many natural substances modulate TLR expression and signalling. For instance, RP105, initially discovered in murine B cells [14], displays several similarities with TLR; it has an extra cellular leucin-rich domain and a TLR-like pattern of juxtamembrane cysteins; its surface expression depends on the cosecretion of a secreted helper protein, in this case, MD1 [15]. However, unlike TLR, RP105 lacks an intracellular domain. Furthermore, the extra cellular domain is a specific TLR4 homologue [16]. It therefore acts as a physiological inhibitor of TLR4 signalling. This was elegantly demonstrated and reviewed elsewhere [16, 17]. In summary, the complex RP105-MD1 interacts directly with the TLR4 signalling complex, preventing its ability to bind LPS. It regulates TLR4 signalling in various immune cells as well as in mice challenged with intraperitoneal *E. coli* LPS. Modulation of the RP105-MD1 complex could help abolish TLR4 overstimulation. Further clinical development is warranted to evaluate a potential role in the treatment of sepsis and associated clinical states. Some of the other natural molecules aimed at controlling TLR effects are listed in Table 1.

Since lipopolysaccharide (LPS) or endotoxin is a specific ligand for TLR4, and because TLR4 expression is increased on human monocytes in healthy volunteers undergoing LPS challenge [18], and in patients with sepsis [19, 20], particular attention has been made to this receptor and signalling pathway. The purpose of this paper is to focus on various drugs interfering with TLR4 expression or TLR4-related intracellular pathway and their potential role as adjunctive therapy in severe sepsis and septic shock, or as modulator of the TLR4-induced inflammatory response (Table 2).

## 2. Antibodies Directed against TLR4 and the TLR4-MD2 Complex

Soluble decoy receptors provide important negative regulatory mechanisms for cytokines and chemokines, and their interaction with their membrane-bound receptor. For instance, increased levels of soluble TNF*α* receptor (sTNFR) are present up to 24 hours after an LPS challenge in healthy volunteers and correlate with the severity of the insult in critically ill patients where low level of sTNFR predicts higher mortality [21]. In mice, Iwami et al. were able to clone a splice TLR4 mRNA that encodes a soluble 20-kDa protein [22]. When expressed in Chinese ovary (CHO)-K1 cells, this protein is secreted in the culture medium. It inhibits LPS-mediated TNF*α* secretion and NF*κ*B activation in a mouse macrophage cell line. Interestingly, LPS stimulation increased the sTLR4 mRNA expression, suggesting a negative feedback to inhibit excessive cytokine production. Any compound able at increasing this natural soluble TLR4 would thus be of potential interest in treating patients with sepsis. 

A specific antibody raised against the ectodomain of TLR4 was recently described [23]. In summary, a chimeric protein composed of the N-terminal half of the mouse TLR4 ectodomain was fused to the Fc domain of human IgG1. In the presence of soluble MD2, this protein binds LPS and inhibits LPS-induced TNF*α* release in whole blood. It was then used to generate high titres of rabbit antimouse TLR4 antibody. These antibodies were able to inhibit response of immune cells exposed to LPS or Gram-negative bacteria in vitro and in vivo. Furthermore, this antibody protects from lethality in mice exposed to endotoxemia or live *E. coli* [23]. 

Another TLR4 antibody was developed [24]. The extra cellular portion of mouse TLR4 was fused with mouse MD-2 via a 15-amino-acid flexible linker. IgG Fc fragments were added to the molecule. This molecule dose-dependently inhibits IL-6 production in RAW 264.7 cells exposed to LPS, and, binds to the surface of Gram-negative bacteria. Depending on the IgG isotype, it also modulates phagocytosis and complement activation. Hence, this molecule could act through 2 distinct mechanisms: on one hand, LPS binding and decreased inflammatory response, and, on the other hand improved bacterial phagocytosis and complement mediated killing [24]. 

Further development is required before these molecules could undergo clinical evaluation. 

## 3. Eritoran or E5564

E5531 is a first generation lipid A analogue, derived from the lipid A structure from the endotoxin of Rhodobacter capsulatus. It blocks LPS in cell culture without any endotoxin-like activity [25]. E5531 protects mice from lethal doses of LPS, and viable *E. coli* infections in combination with antibiotics [25]. It also blocks the endotoxin response in human healthy volunteers exposed to intravenous LPS [26]. Some issues on E5531, such as decreased activity over time in human blood due to interaction with plasma lipoproteins [27, 28], led to the search for second generation LPS antagonist (reviewed in [29]). 

Like E5531, E5564, or eritoran is a synthetic molecule, derived from the nonpathogenic Rhodobacter sphaeroides [30]. The crystal structure of the TLR4-MD2 complex with bound eritoran was recently described, suggesting that eritoran mechanism of action lies within its binding in a large hydrophobic internal pocket in MD2 [31]. Hence, it acts as a LPS antagonist, since it is unable to trigger the intracellular signalling cascade leading to NF*κ*B translocation to the nucleus.

Consequently, eritoran blocks the in vitro production of cytokines in human whole blood [32] and induces a downregulation of intracellular generation of pro-inflammatory cytokines [33]. Pharmacodynamic studies demonstrated a continuous efficacy with every 12 hours intermittent infusion of the drug [34]. 

As for E5531, eritoran reportedly decreases clinical signs, biological parameters, and inflammatory response induced by endotoxin in healthy volunteers [35]. Efficacy of various doses of eritoran, ranging from 50 mcg to 250 mcg, was assessed, while subjects were challenged with 4 ng/kg LPS. All eritoran doses achieved statistically significant reductions in elevated temperature, heart rate, C-reactive protein levels, white blood cell count, TNF*α* and IL-6 levels. In the higher doses groups (>100 mcg/kg), eritoran also statistically blunted the LPS-induced clinical signs such as fever, chills, headache, myalgia, and tachycardia. 

A trend toward decreased mortality was observed in a phase II randomised controlled trial [36]. This study, conducted in North America, recruited 293 patients who were randomised to 3 groups: Eritoran high dose (105 mg), Eritoran small dose (45 mg/d), or placebo. Actually, eritoran at a dose of 105 mg/d administered every 12 hours for 6 days, decreased mortality from 56,3% to 33.3% in patients with high risk of mortality, as assessed by the Acute Physiology and Chronic Health Evaluation II (Apache II) Score. A large ongoing phase III randomised, double-blind, placebo-controlled study is therefore recruiting patients with suspected or proven infection, criteria for the systemic inflammatory response syndrome and at least 1 sepsis-related organ dysfunction. Baseline APACHE II score must range between 21 and 37. Treatment has to be started within 12 hours after the onset of organ failure. We expect the trial to be completed by the end of 2010. 

Eritoran could also modulate sepsis driven organ dysfunction such as cardiac depression and vasodilation, 2 frequent symptoms encountered in severe sepsis and septic shock. Indeed, while the expression of TLR4 on cardiac myocytes is known for years [37], the use of eritoran recently helped identify, in animal models, the role of TLR4 and intra-cellular signalling as one of the mechanism involved in sepsis-related cardiac dysfunction [38]. After 6 hours exposure to LPS, isolated cardiac myocytes from C3H/HeN mice (a normally LPS susceptible strain) develop a reduced sarcomere shortening amplitude and prolonged duration of relaxation. The addition of 2 *μ*g/mL eritoran to the cultured medium leads to a reduced effect of LPS on all monitored contractile parameters. Eritoran further prevents attenuation of contractility observed in LPS treated isolated aortic rings from these mice [39]. Taken together, those data reinforce the idea that this molecule could help treating patient with severe sepsis, beyond its role in preventing cytokine production by immune cells. 

Eritoran could also modulate other noninfectious disease processes, using the TLR4 pathway. Actually, in a model of myocardial ischemia-reperfusion syndrome in C57BL/6 mice, the use of eritoran resulted in smaller infarct, decreased JNK phosphorylation, NF*κ*B translocation, and cytokine production [40]. Because of the well-described increased level of endotoxemia in patients undergoing cardio-pulmonary bypass and the just-mentioned effects of eritoran on the heart and large vessels, eritoran efficacy was assessed in a double-blind, randomised, ascending dose, placebo-controlled trial in patients undergoing cardiac surgery [41]. While no statistically relevant difference could be observed in various inflammatory parameters, no significant safety concern was identified. 

## 4. Resatorvid or TAK 242

TAK 242, or ethyl-(6R)-[N-(2-chloro-4-fluorophenyl] sulfamoyl] cyclohex-1-hene-1-carboxylate, identified by Takeda pharmaceuticals, is a small compound developed to inhibit inflammatory mediators production [42]. It initially was demonstrated to decrease NO and various cytokines production in LPS stimulated mouse macrophages, as well as in a mice endotoxin model [42]. A further study demonstrated its ability to inhibit intracellular signalling, with decreased MAPkinases phosphorylation and I*κ*B degradation, without any interference with LPS binding to TLR4 [43]. Since the effects of ligands to other TLR were not affected, this effect was specific for TLR4. Similar results were obtained using human peripheral blood mononuclear cells (PBMCs), monocytes and macrophages. While the action of TAK 242 in the intracellular domain of TLR4 is known for some times [44], Takashima et al. only recently demonstrated TAK 242 to inhibit TLR4 signalling by direct binding to a specific amino acid (Cys747) in the TLR4-intracellular domain [45]. 

In a mice intraperitoneal endotoxin model, intravenous TAK 242 inhibits the pro-inflammatory response and prevents lethality in a dose-dependent manner [46]. Of importance, treatment up to 2 hours after the LPS challenge results in similar benefits. In an intravenous endotoxemia model using conscious guinea pig, the use of TAK 242 allows better hemodynamic control, decreased level of HMGB-1 and a dose-dependent improved survival [47].

In phase 1 clinical studies in normal healthy subjects given concomitant endotoxin, TAK 242 inhibited the production of cytokines TNF*α*, IL-6 and IL8. Nonclinically significant haemolysis and increases in methemoglobin levels were occasionally observed. A large, multicentre, multinational, randomised, double-blind, placebo-controlled study was initiated in September 2005 (http://clinicaltrials.gov/. NCT00143611). 18-year-old or older subjects with severe sepsis and related respiratory or cardiovascular failure were eligible. The study was ended prematurely after the DSMB determined there was insufficient cytokine suppression in the 150-subject analysis within stage 1 of the study. Another study was planned but unfortunately never started based on business decision (http://clinicaltrials.gov/. NCT00633477). While further development in sepsis patient is unlikely, the potential benefit of TAK 242 in other TLR4 related diseases, such as autoimmune diseases, has to be assessed.

## 5. Chloroquine and Other TLR 9 Antagonists

While the major signalling pathway of LPS lies within its binding to the MD2/TLR4 complex, several reports have indicated that endotoxin may enter immune cells [48] and localize in the Golgi apparatus and other vesicles [49]. This was further confirmed in human PBMC [50]. Therefore, intracellular receptors and medication interfering with those receptors or with intracellular trafficking could be of importance. 

Actually, TLRs that recognize nucleic acids, such as TLR3, 7, 8, and 9, are confined to endocytic compartment where they encounter ligands internalised through receptor-mediated endocytosis or phagocytosis. Upon stimulation of cells, TLR9, for instance, appears to be trafficking from endosome to lysosome where it undergoes proteolytic maturation in an acidic environment to become competent [51]. Asparaginase endopeptidase looks critical for this phenomenon [52]. 

Recently, Plitas et al. demonstrated in a TLR9^−/−^ mice model of cecal ligation and puncture-(CLP-) related peritonitis, an increased bacterial clearance, decreased serum cytokine production and increased granulocytes influx in the peritoneum as compared to wild type animals [53]. Using an inhibitory CpG sequence that blocks TLR9 just before the CLP, they also demonstrated an improved survival in wild type animals. 

Taken together, those data suggest that medication able at blocking TLR9 maturation or signalling could be of interest in sepsis. Actually, chloroquine, a drug used in infectious (malaria) and inflammatory (SLE) diseases, blocks trafficking and, or acidification of the endosome. It is known for years that chloroquine decreases the in vitro response to various pro-inflammatory stimuli such as LPS [53] or CpG oligonucleotide. In vivo, chloroquine protected mice from lethal doses of LPS or CpG through a decrease of proinflammatory cytokine release [54]. Using murine macrophage ANA-1 cells, the authors further demonstrated with chloroquine a decreased expression of TLR4 and 9 mRNA expressions as well as a blockade of NF*κ*B and AP1 activation. Chloroquine demonstrates its positive effects when used prior to the induction of CLP, but also up to 6 hours after [55]. In this experiment, decreased splenic apoptosis was observed, suggestive of a mechanism that improves sepsis-induced immune paralysis. Renal function was also improved. 

Hence, chloroquine may act at 2 different levels: down-regulation of TLR4 expression and interfering with the intracellular trafficking of LPS through its action on TLR9. Of notice, chloroquine also interferes with other TLRs that are internalised and function through endosomal pathway. Whether its actions in polymicrobial sepsis are TLR9 specific or nonspecific has still to be elucidated. 

Because of its excellence tolerance, further clinical development in sepsis and critical care looks promising. 

## 6. Ketamine

Because of its effects on hemodynamic, ketamine, an intravenous anaesthetic agent is widely applied in critical care for induction of anaesthesia or even for maintenance of sedation. Anti-inflammatory effects of ketamine are widely demonstrated in various in vitro animal and human models. The ketamine effects on TLR expression are less known. In a rat model of intravenous LPS stimulation, TLR4 expression and NF*κ*B activation were decreased in the intestine of ketamine-treated animals [56]. Using the same model, the authors demonstrated identical results in the lungs [57]. In a rat model of CLP, treatment with ketamine after the procedure decreased intestine levels of pro-inflammatory cytokines, as well as NF*κ*B activation and TLR4 and 2 mRNA expression, when compared to rats treated with saline [58]. Again, similar results were observed in the lungs, with decreased secretion of pro-inflammatory cytokines, decreased activation of NF*κ*B and decreased TLR2, and 4 mRNA expressions [59]. Doses of ketamine used in these various experiments (up to 10 mg/kg) are far beyond doses used in clinical settings. Mechanisms of action of ketamine were studied in cultured murine macrophage cell line Raw264.7. Not only does ketamine interfere with LPS binding to LBP, but it also decreases phosphorylation of various kinases involved in the TLR4 intracellular signalling [60]. Likewise, ketamine-treated macrophages, stimulated with lipoteichoic acid, a TLR2 agonist, produced less TNF*α* and IL-6. This results from decreased phosphorylation of ERK1/2, an upstream protein kinase for activating inhibitor of NF*κ*B (I*κ*B) kinase (IKK), leading to decreased NF*κ*B translocation to the nucleus [61]. 

Clinical relevance of those results has to be assessed for patients with sepsis or for patients sedated with ketamine. 

## 7. Nicotine

Since the description of the so-called cholinergic anti-inflammatory pathway [62], nicotine and analogues were studied in various cultured cells and animal models of sepsis, pancreatitis, and ischemia-reperfusion syndrome. In humans, transcutaneous nicotine exposure alters the LPS-induced inflammatory response in healthy volunteers [63]. While the nicotinic acetyl-choline receptor, specifically those comprised only of alpha-7 subunits, on myeloid cells are required for this effect [64], the precise intra-cellular mechanism of action is not fully elucidated. Activation of the JAK2-STAT3 pathway and suppression of the NF*κ*B activity at the transcriptional level are implied [65, 66]. Recently, Kox et al. confirmed the reduced cytokine production in human PBMC treated with nicotinic analogues, whatever the stimulated TLR [67]. This effect is likely mediated by JAK2/STAT3 signalling. Interestingly, they also demonstrated with GTS-21, a potent *α*-7 selective partial agonist, modulation of TLR expression after LPS stimulation; TLR2 up-regulation was decreased while TLR4 up-regulation was completely abolished. This further confirms an earlier experiment where nicotine induced a downregulation of TLR4 expression on human monocytes, with or without concomitant LPS stimulation [68]. 

All those data strongly support a potential role for nicotinic agonists to modulate cytokine production as well as toll like receptor expression in severe sepsis and septic shock. Further investigations are required. 

## 8. Opioids

For years, we know that TLRs are expressed in the central nervous system (CNS): while microglia express a wide range of TLRs, astrocytes and oligodendrocytes mainly express TLR2 and 3 [69]. Interestingly, enhanced TLR expression is observed in inflamed CNS tissues. We also know that morphine and opioid derivates display, beyond their role in pain control, important immunomodulatory effects, characterized in animal as well as in human studies (reviewed in [70]). 

TLRs are a key link between the innate immune system and the CNS. Furthermore, several reports demonstrate the involvement of TLR in various types of pain (chronic, neuropathic and inflammatory) as well as in morphine tolerance (reviewed in [71]). Very interestingly, TLR4 was demonstrated to be of particular importance, since select opioids may nonstereoselectively influence its signalling, while having no effects on classical morphine receptors [72]. Indeed, morphine-3-glucuronide, a morphine metabolite with no opioid receptor activity, displays significant TLR4 activity. 

Those data raise at least 2 hypotheses; first, modulation of TLR, in particular TLR4, could be a strategy in the management of chronic pain. Secondly, the use of morphine and other opioids in the critical care setting could interfere with the response to inflammatory stimuli such as LPS. Again, clinical consequences of this warrant further investigation. 

## 9. Statins

Beyond their well-demonstrated lipid lowering effects resulting in clinical benefits in cardiovascular diseases, 3-hydroxy3-methylglutaryl-(HMG-) coenzyme A inhibitors, or statins, display pleiotropic effects. Statins inhibit NF-*κ*B activation and the subsequent pro-inflammatory cytokines such as TNF*α* and IL-6 production. They also blunt endotoxin related activation of cultured human coronary endothelial cells and human PBMC. While these effects are known for years, Methe et al. only recently reported an effect of statins on TLR4 expression [73]. They demonstrated a dose dependent decrease of TLR4 mRNA and protein expression in CD14+ human monocytes incubated in vitro with simvastatin or atorvastatin. They observed a similar effect in vivo, in 12 normocholesterolemic healthy volunteers. Four weeks treatment with atorvastatin 20 mg/d resulted in a 36.2% reduction in TLR4 expression on CD14+ monocytes. Intracellular mechanism of action could include inhibition of protein geranylgeranylation and farnesylation leading to the hypothesis that proteins of the Ras family and the phosphoinositide 3-kinase/protein kinase Akt-pathway are of importance in mediating the TLR4 expression [73]. In accordance with those results, simvastatin 80 mg/d for 4 days decreased the endotoxin-related upregulation of TLR2 and 4, in 20 healthy volunteers exposed to 2 ng/kg intravenous LPS [74]. This expression modulation was also demonstrated in moderate chronic heart failure patients [75]. Interestingly, statins were demonstrated to be most active in reducing the risk of cardiovascular diseases in patients carrying the G allele for TLR [76]. In human embryonic kidney (HEK) 293-CD14-MD2 cell transfected with various TLR4 variants, Hodgkinson and Yee demonstrated that TLR4 variations and statins have an additive inhibitory effect on TLR4-mediated response to LPS, in term of NF*κ*B activation and cytokine production [77]. They further emphasize the role of geranylgernayltransferase and Rho-kinase inhibition to explain the statin intracellular mechanism of action. 

Another hypothesis on the effect of statin on TLR expression is its potential influence on TLR4 membrane trafficking in treated subjects because of altered cholesterol rich membrane domains, as observed in brain plasma membranes [78]. 

The potential of statins as an adjunctive therapy for severe sepsis is currently evaluated in various clinical trials. Actually, a recent meta-analyse suggests that statin treatment may be associated with a beneficial effect in treating and preventing various infections [79]. Because of the presence of heterogeneity and publication bias further randomised trials are required. 

## 10. Vitamin D3 and Analogues

Beyond its important role as a regulator of the calcium-phosphate homeostasis, the hormonally active form of vitamin D displays numerous effects on the immune system [80]. Vitamin D3 and analogues were demonstrated to be protective in a mice model of intraperitoneal endotoxin shock [81]. This positive effect was also demonstrated on coagulation parameters in a rat model of CLP-related sepsis model [82]. Regulation of thromboxane A2 and free radical formation were initially proposed as mechanisms of action [83]. We now know that TLR activation in human monocytes and macrophages leads to an upregulation of the vitamin D receptor (VDR) and the vitamin-D-1 hydroxylase gene expression [83]. These authors also demonstrate that, in presence of vitamin D, this up-regulation leads to increased expression of the cathelicidin mRNA. This cationic antimicrobial peptide is stored in secretory granules and is processed during or after secretion into its mature form, LL-37 [84]. This *α*-helical peptide displays several functions, including killing of pathogens, neutralizing LPS, or acting as a chemo attractant [85]. 

The effect of vitamin D on TLR surface expression is inconstantly reported. On one hand, vitamin D3 was shown to decrease TLR2 and 4 mRNA and protein expression in a time- and dose-dependent fashion in human monocytes [86]. This led to a decreased production of cytokines and tissue factor production as well as a decreased NF*κ*B translocation to the nucleus after LPS or LTA stimulation. Interestingly, the effect of vitamin D3 on TLR expression was VDR-dependent. On the other hand, TLR expression and MD2 expression were not affected by vitamin D3 in cultured human endothelial cells (HMEC) [87]. Nonetheless, vitamin D3 pretreatment resulted in decreased LPS-induced IL-6 and IL-8 production and blocked NF*κ*B activation. 

Taken together, all these data suggest a clear relationship between vitamin D and the TLR pathway and its biological outcomes. Actually, compared to healthy controls, critically ill patients with sepsis had lower levels of serum hydroxyl-vitamin D and LL-37 [88]. It looks therefore logical to assure a normal vitamin D serum level in patients with severe sepsis or septic shock. This should however further be assessed in a randomised trial.

## 11. Other Molecules

Because of their strong antisecretory effects, proton pump inhibitors (PPI) are widely used to treat gastric and duodenal ulcer as well as reflux oesophagitis. They were also reported to display anti-inflammatory and immune properties. For instance, they attenuate polymorphonuclear-dependent gastric mucosal inflammation by interfering with NF*κ*B activation in gastric epithelial cells and vascular endothelial cells [89]. They also modulate the cytosolic concentration of calcium in polymorphonuclear cells. In a study using endotoxin-stimulated 293hTLR4/MD2-CD14 cells, lansoprazole modulated intranuclear transfer of NF*κ*B and stimulated the expression of Suppressor of cytokine signalling-1 (SOCS1), a negative feed back gene involved in excessive LPS stimulation [90]. Therefore, the use of PPIs could modulate the intracellular cascade after TLR4 stimulation. However, those results warrant further investigations in other cell types as well as in vivo. 

Lidocaine, a widely used local anaesthetic, has been reported to attenuate cytokine-induced cell injury and inhibit iNOS expression in activated murine macrophage [91]. To further investigate this latter effect, Lee et al demonstrated that it attenuates the up-regulation of TLR4 expression, NF-*κ*B, and some MAPKinases, in murine macrophages stimulated with LPS [92]. Voltage sensitive sodium channels, if present in macrophages, could be involved in the modulation of TLR and downstream signals modulation. While the use of lidocaine was demonstrated to attenuate acute lung injury in rats exposed to intraperitoneal doses of endotoxin [93], clinical relevance in humans remains to be established. 

Glycine, a *α*-amino acid that acts as an inhibitory neurotransmitter in the central nervous system, also exerts immune-modulating actions via stimulation of glycine-gated chloride channels in immune cells. Glycine inhibits LPS-binding protein (LBP) mRNA expression in the liver mice challenged with LPS, also decreases TLR4 mRNA expression, and decrease activity of NF*κ*B in kupffer cells of those animals [94]. Because of inconsistent result in various animal models, the clinical use of glycine as an immune-modulating agent in sepsis remains to be elucidated. 

## 12. Conclusions

The TLR4 signalling pathway leading to lipopolysaccharide-mediated NF-*κ*B activation constitutes an important therapeutic target for sepsis therapy. Various molecules are involved in TLR4 membrane regulation and could behave as new adjuvant therapies able to weaken the deleterious effects of exaggerated host response to infection. Most of those are not yet exploited and additional laboratory and clinical investigations are required to confirm their expected influence. Many studies have documented capacities of new drugs to regulate TLR4 signalling and expression. For most of them, mechanisms underlying this action still need to be straighten out. Moreover, clinical implications remain to be corroborated, especially for those medications already used for other indications, such as ketamine. A better knowledge of TLR4 regulation molecules will be crucial to control host to infection reaction and avoid the detrimental consequences of sepsis. Among the drugs looking promising, eritoran, a lipid A analogue, is undergoing a large phase III clinical trial. 

## Figures and Tables

**Table 1:** Some of the natural inhibitors of TLR4 signalling.

Level of action	Natural TLR4 signaling inhibitor	
Extracellular	Soluble CD14	sCD14
	Soluble MD2	sMD2
	Soluble TLR4	sTLR4

Membrane	Receptor RP105	RP 105
	TNF-Related Apoptosis Inducing Ligand-Receptor	TRAIL-R
	Receptor ST2	ST2

Intracytoplasmic		

(i) MyD88	Short form of MyD88	MyD88s
	Single Immunoglobulin Il-1 Receptor-related molecule	SIGIRR
(ii) IRAK	Interleukin-1 receptor associated kinase M	IRAK M
	Monarch 1	
	Toll Interacting Protein	TOLLIP
(iii) TRAF-6	A20	
	*β*-arrestin	
(iv) P38 and JNK kinases	Phosphatidyl inositol 3 kinase	PI3K
(v) NF*κ*B	A20-binding inhibitor of NF*κ*B activation	ABIN-3
	p50 and Mel B dimers	
(vi) Cytokine secretion	Suppressor of cytokine secretion 1	SOCS1

**Table 2:** Mode of action of various molecules targeting TLR4.

Molecules interfering with TLR4 and TLR4-mRNA expression
(1) Chloroquine
(2) Ketamine
(3) GTS21 (nicotinic analogue)
(4) Statins
(5) Vitamin D3 (?)
(6) Lidocaine
(7) Glycine

Molecules interfering with TLR4-related intracellular signalling pathway
(1) Eritoran (E5564)
(2) Resatorvid (TAK242)
(3) Ketamine
(4) Opioids
(5) Vitamin D3 (through its action on LL37)
(6) Lansoprazole (through its action on SOCS1)
